# Micro RNAs in Regulation of Cellular Redox Homeostasis

**DOI:** 10.3390/ijms22116022

**Published:** 2021-06-02

**Authors:** Sylwia Ciesielska, Izabella Slezak-Prochazka, Patryk Bil, Joanna Rzeszowska-Wolny

**Affiliations:** 1 Department of Systems Biology and Engineering, Faculty of Automatic Control, Electronics and Computer Science, Silesian University of Technology, 44-100 Gliwice, Poland; Patryk.Bil@polsl.pl (P.B.); Joanna.Rzeszowska@polsl.pl (J.R.-W.); 2 Biotechnology Centre, Silesian University of Technology, 44-100 Gliwice, Poland; Izabella.Slezak-Prochazka@polsl.pl

**Keywords:** reactive oxygen and nitrogen species, miRNAs, ROS/RNS neutralization, ROS scavenging enzymes, ROS producing enzymes, ROS/miRNA mutual regulation

## Abstract

In living cells Reactive Oxygen Species (ROS) participate in intra- and inter-cellular signaling and all cells contain specific systems that guard redox homeostasis. These systems contain both enzymes which may produce ROS such as NADPH-dependent and other oxidases or nitric oxide synthases, and ROS-neutralizing enzymes such as catalase, peroxiredoxins, thioredoxins, thioredoxin reductases, glutathione reductases, and many others. Most of the genes coding for these enzymes contain sequences targeted by micro RNAs (miRNAs), which are components of RNA-induced silencing complexes and play important roles in inhibiting translation of their targeted messenger RNAs (mRNAs). In this review we describe miRNAs that directly target and can influence enzymes responsible for scavenging of ROS and their possible role in cellular redox homeostasis. Regulation of antioxidant enzymes aims to adjust cells to survive in unstable oxidative environments; however, sometimes seemingly paradoxical phenomena appear where oxidative stress induces an increase in the levels of miRNAs which target genes which are supposed to neutralize ROS and therefore would be expected to decrease antioxidant levels. Here we show examples of such cellular behaviors and discuss the possible roles of miRNAs in redox regulatory circuits and further cell responses to stress.

## 1. Introduction

MicroRNAs (miRNAs) are a class of short, single-stranded non-coding RNAs that repress gene expression at the post-transcriptional level. MiRNAs associate with one of the Argonaute proteins to form an RNA-induced silencing complex (RISC) that binds mainly to the 3′untranslated regions (UTRs) of their target messenger RNAs (mRNAs) to inhibit their translation or induce their degradation [1,2]. MiRNAs target mRNAs by complementary base-pairing, and their region crucial for this miRNA-mRNA interaction is the “seed” sequence, namely a heptamer sequence mostly situated at positions 2–7 from the miRNA’s 5′-end. The majority of protein coding genes is thought to be under control of miRNAs [3,4] and miRNAs are involved in virtually all biological processes including proliferation, differentiation, and programed cell death. In addition, miRNAs have been causally linked to many pathological conditions including diabetes [5], cardiovascular disease [6], autoimmune disorders, and cancer.

Redox status is defined as the potential to donate or receive electrons for biochemical processes and cells achieve a balance between oxidants, including free radicals, and antioxidants. Cells maintain redox balance through generation and elimination of reactive oxygen species (ROS) and reactive nitrogen species (RNS) [7,8]. ROS may be generated by exogenous or endogenous sources; exogenous sources encompass ionizing and non-ionizing radiation, drugs, pollutants, food, ultrasound, xenobiotics, and toxins [7,9,10], and endogenous ROS sources are cellular organelles with high oxygen consumption such as mitochondria, peroxisomes, and endoplasmic reticulum [11]. RNS include nitric oxide (NO), peroxynitrite (ONOO^−^), and nitrogen dioxide (NO_2_). The main representatives of cellular ROS are superoxide radical (O_2_^•−^), hydroxyl radical (^•^OH), singlet oxygen (^1^O_2_), and hydrogen peroxide (H_2_O_2_) which are formed in significant amounts as toxic byproducts in the reactions of the mitochondrial respiratory chain [11]. Significant amounts of O_2_^•−^, H_2_O_2_, ^•^OH and NO can be also produced in the respiratory pathway of peroxisomes, where electrons from different metabolites reduce O_2_ and energy is released in the form of heat [12]. The major metabolic process producing H_2_O_2_ in peroxisomes is β–oxidation of fatty acids and the central role of peroxisomes is to reduce H_2_O_2_ [11,12]. MiRNAs can target and influence the level of enzymes responsible for production and scavenging of ROS. In this review we summarize the cellular systems that regulate production and scavenging of ROS and RNS and describe the role of miRNAs as modulators of antioxidant effects. We focus on miRNAs that directly target and regulate enzymes responsible for production and neutralization of ROS and RNS and the possible role of these miRNAs in cellular redox homeostasis, and also on the influence of ROS on miRNA levels and on mutual regulation of ROS and miRNAs.

## 2. ROS/RNS Production and Neutralization in Cells

**Figure 1:**
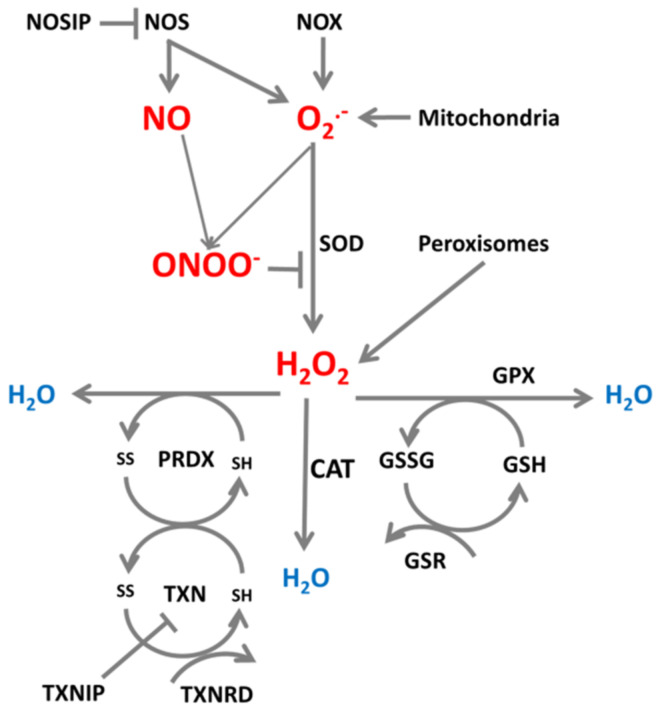
ROS/RNS production and neutralization. Cellular superoxide (O_2_^•−^) and nitric oxide (NO) and produced and further converted to peroxynitrite (ONOO^-^) by reaction of NO with O_2_^•−^, or to H_2_O_2_ by superoxide dismutase (SOD). H_2_O_2_ can be further neutralized to H_2_O by catalase (CAT), glutathione peroxidase (GPX) and peroxiredoxins (PRDX) which are reduced by thioredoxins (TXN). TXN is reduced by thioredoxin reductase (TXNRD) and glutathione (GSH) is reduced by glutathione-disulfide reductase (GSR).

Enzymes producing and reducing ROS/RNS are important participants in systems which maintain a physiological intracellular redox environment, and an example of such a system which neutralizes superoxide is presented in Figure 1.

NADPH oxidases (NOX) are enzymes which by design generate ROS and therefore regulate numerous redox-dependent signaling pathways which influence cell differentiation, proliferation, apoptosis, and embryonic development [13]. Especially, NOX2 and NOX4 are recognized for their role in ROS generation; NOX2 produces O_2_^•−^ whereas NOX4-expressing cells contain detectable levels of H_2_O_2_ rather than O_2_^•−^ [14]. Cells may produce O_2_^•−^ not only by enzymes from the NOX family [10] but in some circumstances by nitric oxide synthases (NOS) [15], a group of three isoenzymes: neuronal (NOS1), inducible (NOS2) and endothelial NOS (NOS3) [16]. The active NOS proteins consist of a reductase domain and an oxidase domain, coupled together by the calcium-dependent enzyme calmodulin [17]. An important property of NOS is their ability to switch between production of superoxide radical or nitric oxide which depends strictly on availability of the cofactor tetrahydrobiopterin (BH4) and its oxidized form dihydrobiopterin (BH2) [18]. The substrate for production of nitric oxide (and l-citrulline) by NOS is l-arginine, whose level is regulated by enzymes of the urea cycle [17,19].

To counteract increases in ROS caused by environmental factors, cells use a variety of antioxidants which create anti-oxidative systems. Antioxidants are special molecules which can convert and neutralize ROS and regulate their levels by various pathways [9], and the main elements of such systems are small molecules which easily exchange electrons and whose oxidized and reduced states create redox pairs. Examples are glutathione/glutathione disulfide (GSH/GSSG), oxidized/reduced nicotinamide adenine dinucleotide (NAD+/NADH), and nicotinamide adenine dinucleotide phosphate (NADP+/NADPH) [20,21]. To keep the proper concentrations of these molecules in their antioxidant form, cells contain enzymes which are able to reduce them. The ratio of oxidized to reduced form of glutathione is often used as an indicator of the redox state of the cell [20].

Superoxide radical is converted to H_2_O_2_ by superoxide dismutase (SOD) [22,23]. Nitric oxide competes for superoxide with SOD, and superoxide together with nitric oxide can form peroxynitrite, which inhibits SOD. SOD is an element of an antioxidant system protecting cells from harmful effects of the free radical O_2_^•−^ [22] by converting O_2_^•−^ to H_2_O_2_ and oxygen. O_2_^•−^ can be neutralized by three isoforms of SOD, by CuZnSOD (SOD1) in the cytosol, by MnSOD (SOD2) in mitochondria, and by extracellular SOD (SOD3) in the extracellular space [22]. Hydrogen peroxide (H_2_O_2_) is believed to be the most toxic ROS; it can pass freely through membranes and may induce direct breakage of the phosphodiester backbone of DNA [24]. H_2_O_2_ can be converted to OH in the presence of copper and iron [25,26] or may be neutralized to H_2_O by catalase (CAT), an antioxidant present in almost all aerobic organisms [27], by interaction with glutathione peroxidase (GPX) which converts glutathione to its oxidized GSSG form [20,28], and by enzymes of the peroxiredoxin family (PRDX) which, by reducing H_2_O_2_, become oxidized [29,30]. Catalase is one of the oldest antioxidant enzymes, usually classified into three types: typical catalases present in aerobically respiring organisms, catalase-peroxidases present in in fungi, archebacteria and bacteria, and manganese catalases present exclusively in bacteria. Typical catalases are divided into three subgroups, and catalase from the third group is present in archebacteria, fungi, protists, plants, and animals; human catalase belongs to this group [31]. Catalase is present mainly in peroxisomes of most cells (especially liver cells) and in the cytoplasm (erythrocytes), it is absent in mitochondria although reported to be present in mitochondria of rat heart [31,32]. In addition to neutralization of H_2_O_2_, catalase decomposes peroxynitrite and oxidizes nitric oxide to nitrite [31,32]. Peroxiredoxins, or more precisely thioredoxin peroxidases, are widely spread thiol-specific antioxidant enzymes which constitute up to 1% of total proteins in some organisms [29] and serve as antioxidants to H_2_O_2_ and ONOO^-^, and can reduce even more than 90% of cellular peroxides. There are six members of the peroxiredoxin family, PRDX1, PRDX2, and PRDX6 localized in the cytosol, PRDX3 localized in mitochondria, PRDX4 localized in the extracellular space, and PRDX5 in mitochondria and peroxisomes [30]. We showed previously that for H_2_O_2_ neutralization different types of cells may activate different pathways, using the most effective pathway through catalase, peroxiredoxins, or glutathione peroxidase [33,34].

To keep the whole system functioning, oxidized antioxidant molecules must be reduced once again and this is the role of glutathione reductases (GSR) [20] and thioredoxins (TXN) [35]. Thioredoxins are small redox proteins which are reduced depending on NADPH by thioredoxin reductase. They serve as donors of electrons and reduce cysteine groups on proteins. Thioredoxin together with thioredoxin reductase constitutes a very important element of the peroxiredoxin and glutathione peroxidase antioxidant systems [35]. Peroxiredoxins, thioredoxins, and glutathione contain cysteines with thiol groups that exist in the reduced (SH) or oxidized (S-S) form. Peroxiredoxins become oxidized when reducing H_2_O_2_, and then are reduced by thioredoxins which become oxidized. To keep the system working, oxidized thioredoxins are reduced by thioredoxin reductases (TXNRD), essential elements of the thioredoxin system, to restore their enzymatic activity [36]. The electrons needed for these reduction reactions originate from reduction reactions permanently ongoing during metabolic reactions [30,36]. Additional regulators of these systems are nitric oxide synthase interacting protein (NOSIP) which interacts with NOS and limits NO production [37], and thioredoxin interacting protein (TXNIP) which modulates the action of the thioredoxin-thioredoxin reductase system and inhibits the antioxidative function of thioredoxin, resulting in accumulation of ROS and cellular stress [35].

## 3. Roles of ROS and RNS in Cellular Processes

ROS are important regulators of many cell functions including proliferation and cell death [38,39]; low levels usually promote cell growth whereas higher concentrations lead to death or senescence [40,41,42]. For example, low doses of O_2_^•−^ stimulate proliferation in leukemia and vascular smooth muscle cells (VSMCs) [43,44]. H_2_O_2_ itself in low concentrations can promote growth of fibroblasts but increased concentrations cause apoptosis [45,46]. A growth-stimulating effect of H_2_O_2_ was also observed in yeast and in bacteria [47,48].

Cell survival can be affected through modification of redox-sensitive signaling proteins connected to survival through signal transduction pathways such as MAPK, PI3K/Akt, or p53, through transcription such as NF-κB, Ap-1, or Nrf-2, or through execution such as caspases, Bcl-2, or cytochrome c [49]. Among interesting is the transcription factor Nrf-2, responsible for regulating expression of many redox-related enzymes including those related to glutathione synthesis and regeneration (GCLC, GCLM, GSS), SOD1, CAT, and also many redox cycling enzymes (e.g., TXN, PRDX) [50]. Biogenesis of miRNA is affected by cellular redox status so changes in Nrf-2 activity may impact miRNA levels, but Nrf-2 expression and activity is regulated by miRNAs which indicates that there is an intricate codependent relationship among Nrf-2, ROS and miRNAs [51]. Some miRNAs, like miR-27a, miR-142-5p, miR-144, and miR-153 downregulate Nrf-2 protein levels but directly targeting Nrf-2 mRNA, but other miRNAs, e.g., miR-200a, target Nrf-2 inhibitor (Keap1) and lead to increased expression of Nrf-2 [51]. The influence of ROS on cell viability, proliferation and apoptosis was also noted in the studies of Kitamoto et al., which show that inhibition of NOX2 and subsequently of ROS promote apoptosis, whereas ROS production by NOX2 increase cell growth in osteosarcomas [52]. Thioredoxin overexpression was found to increase cancer cell growth and inhibit apoptosis [53]. Proliferation can be also affected through redox regulation of chromatin remodeling, which affects death/survival signals at the transcriptional level. Postranscriptional modifications such as phosphorylation of death/survival signaling proteins can be also mediated by redox changes [49]. ROS levels which define cellular redox state affect the oxidation of cysteines in proteins which control progression through the cell cycle [54]. Changes in ROS/RNS levels induce changes in redox status which can influence reactions of oxidation, nitrosylation, and nitration of cellular proteins [55]. For example, an increase in oxidative state may cause activation of cytochrome c and then of caspases which leads to apoptosis [49]. H_2_O_2_-mediated oxidation of cysteines in Cdc25C, PTEN, and LMW-PTP leads to formation of inter-cysteine disulfide bonds and results in inactivation of these phosphatases, which in turn affects cell cycle progression through G2/M and cell survival and growth [56]. S-nitrosylation, a modification of proteins by NO that depends on the cellular localization of NOS, directly alters the activity of NF-κB, an enzyme that regulates activation of genes related to inflammatory responses, apoptosis, cell adhesion, differentiation, and proliferation [57]. Similarly, nitration of tyrosine by ONOO^−^ is also related to cellular localization of NOS, and affects the activity and promotes degradation of proteins such as MnSOD, P53 (tumor suppressor protein), and IκBα (NF-κB inhibitor protein) [58].

## 4. Role of miRNAs in Regulation of ROS/RNS-Producing Enzymes

**Figure 2:**
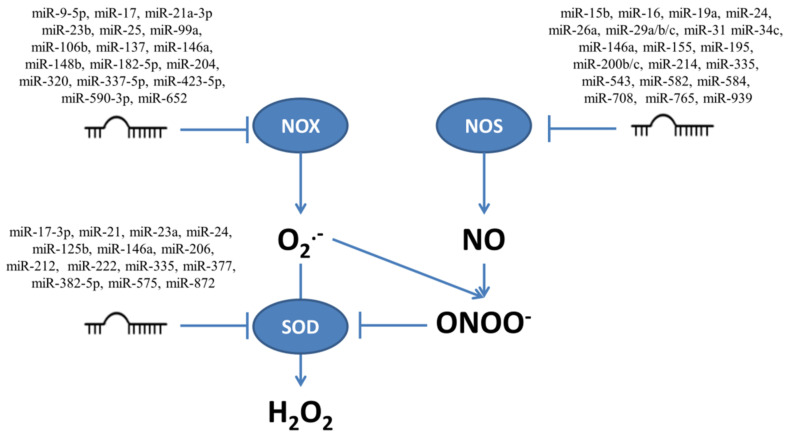
The multiple miRNAs which target enzymes responsible for production (NOX and NOS) and neutralization of superoxide (O_2_^•−^) and nitric oxide (NO), together with superoxide dismutase (SOD) which converts O_2_^•−^ to H_2_O_2_.

The mRNAs for enzymes involved in production and neutralization of ROS and RNS presented in Figure 1 contain sequences which are targeted by multiple miRNAs. Figure 2 shows those miRNAs which target transcripts for enzymes connected to ROS/RNS production, such as NOX and NOS, and to conversion from O_2_^•−^ to H_2_O_2_ such as SOD. The influence of miRNAs on the targeted enzymes has been confirmed experimentally, as illustrated below in examples focusing on miRNAs which influence proliferation and/or apoptosis modulated by ROS/RNS levels.

### 4.1. NADPH Oxidases

Several miRNAs (miR-34a, miR-320, and miR-652) affect ROS production by targeting NOX transcripts. Overexpression of miR-320 in ischemic cerebral neurons reduces NOX2 levels and indirectly increases SOD, CAT, and GPX levels and NOX2 overexpression counteracts these effects; miR-320-mediated NOX2 inhibition causes reduced ROS levels, resulting in induced proliferation and inhibited apoptosis [59]. A second miRNA that impairs ROS production by inhibiting NOX2 expression is miR-652. Overexpressed miR-652 protects brain tissues of rats with middle cerebral artery occlusion (MCAO) from apoptosis, as shown by decreased caspase-3 activity [60]. Accordingly, NOX2 was identified as a positive regulatory target of miR-34a in A172 glioma cells, where its overexpression induced apoptosis and decrease cell viability through enhanced NOX2 and ROS production [61].

NOX4 is targeted by miR-23b, miR-99a, miR-137, miR-182-5p, miR-423-5p, and miR-590-3p [62,63,64,65]. MiR-23b can protect GABAergic neurons from cell death and its increase induced TXNL1 and GPX3 gene expression [62]. Overexpression of miR-423-5p in mouse podocyte cells inhibited ROS generation, enhanced cell viability, and suppressed apoptosis [63]. By targeting NOX4, miR-99a reduced NOX4 levels and also migration and invasion in lung adenocarcinoma and inhibited xenograft growth [64]. In prostate cancer cells overexpression of miR-137 inhibited NOX4, suppressed proliferation, and promoted apoptosis [65]. Overexpression of miR-182-5p increases viability and proliferation and reduces apoptosis in H_2_O_2_-treated human lens epithelial cells through regulating NOX4 and p38 MAPK signaling and can suppress H_2_O_2_-induced oxidative stress through restoring SOD and GPX activity [66]. Downregulation of miR-590-3p increased pyroptosis through its targets NLRP1 and NOX4 and activation of the NOX4/ROS/TXNIP/NLRP3 pathway in human retinal microvascular endothelial cells, and NOX4 overexpression, caused increased production of TXNIP [67].

Additionally, transcripts for NOX enzymes are targeted by several miRNAs, yet no connection to apoptosis or cell growth is observed. NOX-targeting miRNAs include miR-9-5p in fibroblasts [68], miR-17 in human microglial cells [14], miR-21a-3p in a mouse endothelial cell tumor model [69], miR-25 in bone marrow mesenchymal stem cells [70] and primary cardiomyocytes [71], miR-99a in LO2 hepatocytes [72], miR-106b, miR-148b and miR-204 in macrophages [73], miR-146a in human aortic endothelial cells [74], and miR-337-3p in tendon-derived stem cells [75].

### 4.2. Nitric Oxide Synthases

NOS expression can be regulated by different sets of miRNAs. NOS3 expression is down-regulated by miR-155 in VSMCs resulting in accelerated migration and proliferation, in human leukemia monocytes, and in human umbilical vein endothelial cells (HUVECs) where miR-155 has a pro-apoptotic effect [76,77,78,79]. MiR-335 targets the 3′-UTR of NOS3 mRNA in trophoblast cells and prostate cancer, and may significantly decrease the ability to migrate of these tumor cells [80,81]. MiR-543 and miR-584 in trophoblast cells and miR-335 in prostate cancer cells down-regulate NOS3 expression and reduce their migratory capability [80,81]. The effects of miR-24, which targets NOS3 mRNA, vary depending on the cell line [82]; in endothelial cells it is related to decreased proliferation and increased apoptosis, but in contrast in mouse cardiac fibroblasts and cardiomyocytes it leads to lower apoptosis [82]. In endothelial cells miR-200c targets NOS3 and many other enzymes, including PRDX2, which results in lowered NO production and decreased H_2_O_2_ neutralization leading to apoptosis [83]. In HUVECs, miR-31-5p targets NOS3 mRNA, which results in decreased proliferation and migration of endothelial cells under inflammatory conditions [84]. NOS3 is also targeted by other miRNAs including miR-195 and miR-582 in human microvascular endothelial cells [85], miR-15b, miR-16 and miR-30b [86] and miR-200b [87] in HUVECs, miR-214-3p in human renal epithelial cells [88], but their effect on cell survival has not been reported. No effect on cell survival was seen when NOS1 was inhibited by miR-31 in myoblasts [84] or atrial myocytes [89], by miR-34c or miR-708 in myoblasts [90], by miR-146a in Caucasian prostate adenocarcinoma or human glioblastoma astrocytoma [91,92], or when NOS2 was inhibited by miR-939 in primary human hepatocytes [93], by miR-29a/b/c in skeletal muscle cells [94], or by miR-26a-5p in human osteoarthritis chondrocytes [95]. NOSIP can inhibit the enzymatic activity of NOSs, and is down-regulated by miR-372 in human neural stem cells [96].

Data collected for different miRNAs targeting NOX and NOS suggest that these two enzymes may regulate cell survival through generation of ROS/RNS and exhibit opposite effects connected with both pro-survival and pro-apoptotic signals. In Table 1, Table 2 and Table 3 the term ‘pro-survival’ includes increase of proliferation and decrease of apoptosis and ‘pro-apoptotic’ the opposite. Reduction of NOX by miRNAs and subsequently lower levels of ROS, inhibited apoptosis and increased proliferation (white rows); however, for lung adenocarcinoma and prostate cancer (grey rows) the effect was opposite. NOX reduction by miRNAs induced apoptosis and inhibited proliferation and tumor growth. The data in Table 1 (grey rows) concerns mainly cancers, however this response is not related only to cancer, and NOX4 protected vascular function in VSMCs [97].

Reduction of NOS by the same miRNA can lead to vastly different survival in different cell types: VSMCs, cardiomyocytes and cardiac fibroblasts express pro-survival behavior when their NOS3 expression is reduced by miRNAs, while in HUVECs the response to the same miRNAs is opposite. This may be related to the ability of NOS’s to switch between production of NO and O_2_^•−^, which is sensitive to a cell’s redox environment, and thus inhibition of NOS by the same miRNA may lead to production of different types of ROS in different cell types.

## 5. MiRNAs Participating in Regulation of H_2_O_2_ Level

**Figure 3:**
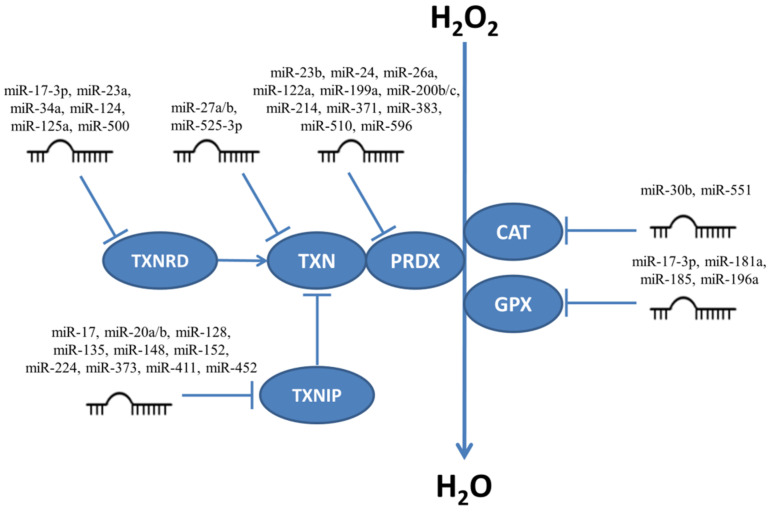
MiRNAs targeting enzymes responsible for neutralizing H_2_O_2_. CAT, catalase; GPX, glutathione peroxidase; PRDX, peroxiredoxin; TXN, thioredoxin; TXNRD, thioredoxin reductase; TXNIP, thioredoxin interacting protein.

Enzymes responsible for H_2_O_2_ neutralization are targeted by many miRNAs (Figure 3) and thus influence the levels of H_2_O_2_ in cells. Below we give examples of experiments which focus on miRNAs influencing proliferation and/or apoptosis.

### 5.1. Regulation of Superoxide Dismutases by miRNAs

MnSOD (SOD2) is targeted by several miRNAs including miR-17-3p, miR-23a, miR-146a, miR-212, miR-222, miR-335, miR-382-5p, and miR-575 [98,99,100,101,102,103,104,105,106]. In prostate cancer cells miR-17-3p targets MnSOD, GPX2, and TXNRD2 leading to ROS accumulation [98]. Upregulation of miR-17-3p sensitized these cells to ionizing radiation via MnSOD, TXNRD2 and GPX [99]. Similarly, enhanced cell death occurs upon miR-17-3p-mediated inhibition of MnSOD and TXNRD2 in human retinal pigment epithelial cells (ARPE-19) [100]. Inhibition of MnSOD by miR-23a lead to apoptosis in cardiomyocytes [101] and by miR-575 in villi cells [102]. Inhibition of miR-146a reverses the decrease of viability in H_2_O_2_-treated rat adrenal gland PC12 cells. Overexpression of miR-212 inhibits migration and invasion in vitro and formation of intrahepatic and pulmonary metastases in vivo in colorectal cancer cells through targeting MnSOD [103]. MiR-335 inhibition significantly reduces ROS levels and its overexpression leads to senescence in mesangial cells. Such behavior can be connected to both miR-335 which targets SOD2 and to miR-34a which targets TXNRD2 [107]. Augmented oxidative stress in mesangial cells was observed when miR-377 is overexpressed [108].

In contrast to the enhanced apoptosis described above after SOD2 inhibition by miR-222, resulted in inhibition of apoptosis in oral tongue squamous cell carcinoma was observed and cell invasion was decreased, possibly through regulation of MMP1 expression [104]. Similarly, SOD2 was likewise identified as a target of miR-222 in cardiomyocytes [105]. In line with this, a decrease in SOD2 and further ROS accumulation was reported after overexpression of miR-382-5p in primary myelofibrosis CD34+ cells, linked to deregulation of the TGF-β1/miR-382-5p/SOD2 pathway [106]. ROS overproduction contributed to enhanced oxidative stress and inflammation. MiR-382-5p overexpression increased proliferation of CD34+ cells while its inhibition reduced oxidative stress and decreased cell proliferation of CD34+ cells [106].

Another miRNA which markedly influences superoxide and hydrogen peroxide metabolism cells is miR-21, which targets SOD3 and through TNFα indirectly attenuates levels of SOD2 in human bronchial epithelial. Levels of H_2_O_2_ were lower than those of O_2_^•−^ after introduction of miR-21 than in control, non-treated cells, for both irradiated and unirradiated cells. MiR-21 overexpression caused a significant increase in colony formation compared both to control cells with a normal level of miR-21 and to unirradiated cells with overexpressed miR-21 [109].

Additionally, SOD enzymes are targeted by several miRNAs, although neither cell growth nor apoptosis are affected; miR-24, miR-125a-3p and miR-872 in Sertoli cells [110] and miR-206 in primary mouse tracheal epithelial cells [111].

Excessed levels of ROS cause cell death or senescence, and the superoxide dismutases can affect cell survival (Table 2) However, it seems that these effects are cell-type specific.

### 5.2. Regulation of Catalase, Glutathione Peroxidases, Peroxiredoxins and Thioredoxin System Enzymes by miRNAs

Catalase transcripts are targeted by miR-30b and miR-551[113,114]. The level of miR-30b was shown to increase after H_2_O_2_ treatment and to bind to the 3′UTR of catalase mRNA causing decrease of protein levels in ARPE-19 cells [113,114]. Catalase expression is also inhibited by miR-551b in human lung cancer cells and the miR-551b/CAT pathway can be involved in acquired apoptosis resistance and chemoresistance through interaction with MUC1 [115].

Three of the eight members of the GPX family have been identified as targeted by miRNAs. The transcript of GPX1 is targeted by miR-181a and can reduce H_2_O_2_-induced apoptosis and ROS production when inhibited in cardiomyocytes [116]. GPX1 is also linked to impaired oxidant response in endothelial cells and associated with miR-185 upregulation by which is directly targeted [117]. MiR-214, targets GSR whose inhibition leads to induction of oxidative stress in liver cells [118]. In addition to SOD2, miR-17-3p also targets GPX2 in prostate cancer cells [98,99]. GPX3 is targeted and downregulated by miR-196a which is overexpressed in non-small-cell lung carcinoma (NSCLC) cancers, leading to attenuation of tumorigenicity and cancer cell growth through upregulation of GPX3. Development of NSCLC cells may be promoted by activation of the JNK pathway through downregulation of GPX3 [119].

The PRDX family members can be targeted by multiple miRNAs. PRDX1 is a direct target of miR-510 and miR-596. Overexpression of miR-510 leads to increased cell growth, migration, invasion and colony formation in breast cancer cells, possibly through activation of the Akt signaling pathway. Treatment of breast cancer cells expressing miR-510 with H_2_O_2_ led to increased cell death [120]. Overexpression of miR-596a can suppress cell proliferation, migration, and invasion in gastric cancer [121].

PRDX2 can be inhibited by miR-122 and miR200b/c. MiR-122a overexpression inhibits cell growth and induces apoptosis in hepatocellular carcinoma through direct inhibition of PRDX2 [122]. MiR-200c is involved in radiosensitivity of lung cancer cells by direct regulation of oxidative stress; cells overexpressing miR-200c are more sensitive to radiation and show significantly increased ROS levels and p21 expression. PRDX2 promotes p21 upregulation in H460 lung cancer cells [123]. PRDX2 is also targeted by miR-200b, which suppress growth, invasion and metastasis in colorectal cancer and is connected to enhanced chemotherapeutic resistance through disruption of the c-Myc/miR-200b-3p/PRDX2 regulatory loop [124].

PRDX3 is a direct target of miR-23b, miR-26a-5p, and of miR-383. Inhibition of both miR-23b [125,126,127] in prostate cancer and leukemia and miR-26a-5p in leukemia [116] can scavenge excessive levels of ROS by increase of PRDX3 gene expression. MiR-383 overexpression decreased cell growth and increased apoptosis through negative regulation of PRDX3 in medulloblastoma [127].

PRDX6 can be regulated by miR-24-3p, miR-214, miR-199a, and miR-371-3p. MiR-24-3p inhibited cell growth, migration, and invasion and promoted apoptosis in gastric cancer cells and overexpressed PRDX6 reversed this effect [128]. Overexpression of miR-199a-3p enhanced proliferation of leukemic cells [129]. MiR-214, which targets PRDX6, was elevated after ionizing radiation and PRDX6 knockdown increased apoptosis after radiation in rat skin [130]. PRDX6 is also targeted by miR-371-3p, whose overexpression in a PC9 xenograft mouse model moderately reduced growth rate [131].

TXN1 was targeted by miR-525-3p and this miRNA appeared to be an important radiosensitivity regulator in EA, HeLa, RPE, and U2-OS cells exposed to ionizing radiation. Unexpectedly, the increase of miR-525-3p promoted survival of these cells after irradiation [132]. MiR-27a/b targets TXN2, whose knockdown can inhibit efficient cell growth in cells infected with adenovirus [133].

One of the miRNAs which target TXNRD1 transcripts is miR-23a/b which is involved in skeletal muscle differentiation, and TXNRD1 depletion reduces myoblast growth [134]. Overexpression of miR-124 repressed TXNRD1 and decreased the surviving fraction of radiation-resistant lung cancer cells. Downregulation of miR-124 mediated radiation resistance through targeting TXNRD1 [135] whose 3′-UTR is also targeted by miR-125a-5p, and this downregulation improves TXNRD1’s antioxidant function in endothelial cells and H_2_O_2_ treatment can inhibit miR-125a [136]. Overexpression of miR-125a-5p in head and neck cancer decreased the surviving fraction after irradiation [137]. Regulation of the *TXNRD1* gene can be also influenced by miR-125b-5p in hepatocellular carcinoma, where the level of miR-125b-5p is reduced; miR-125b-5p inhibited cell proliferation, migration, and invasion [138]. MiR-500-5p in breast cancer, influence oxidative stress response and cell survival through targeting the *TXNRD1* and *NFE2L2* genes [139].

TXNRD2 is targeted by MnSOD-targeting miRNAs including miR-17-3p (described in the Chapter on SOD) [98,99,100]. MiR-34a mimics induce a premature senescence phenotype in young mesangial cells [107]. Thounaojam et al. showed that miR-34 can target TXNRD2 transcripts but not those of SOD2, and miR-34a promotes senescence of human retinal microvascular endothelial cells (HuRECs) [140].

Upregulation of the TXNIP gene can increase ROS production and can be targeted by miR-17 in myocardial cells of diabetic mice. The high glucose level in diabetes decreases miR-17 levels and induces apoptosis [141]. MiR-20a is highly expressed in rheumatoid arthritis [142] and miR-20b in HUVECs [143], and they subsequently silence TXNIP. These miRNAs can enhance cell viability and inhibit senescence [142,143]. In BV2 microglial cells miR-152 overexpression caused a decrease in neuronal cell death [144]. Luciferase reporter assays confirmed that miR-128 targets TXNIP transcripts in pancreatic beta cells [145]. A miR-135a mimic reduced levels of apoptosis in myocardial cells of diabetic mice [146] and a similar effect was observed with miR-148a in alcoholic liver disease [147]; alcohol can decrease miR-148a expression in hepatocytes and subsequently TXNIP is overexpressed. It can induce hepatocyte pyroptosis [147]. MCF7 cells transfected with pre-miR-373 showed an increase of invasiveness and metastasis but not of proliferation [148]. MiR-224, another miRNA identified as targeting TXNIP, promotes pancreatic cancer cell proliferation and migration, elevating levels of HIF1α by targeting TXNIP independently of TXN and ROS [149]. MiR224/452 is involved in melanoma progression through suppression of TXNIP, and its overexpression causes enhancement of migration and invasion. miR-224/452-mediated downregulation of TXNIP is required for E2F1-induced EMT and invasion [150]. In addition, miR-411-5p overexpression in NSCLC cells positively influences cell proliferation and migration and decreases apoptosis through targeting both TXNIP and SPRY4 mRNAs [151].

Inhibition of antioxidant enzymes increases ROS levels and intuitively should negatively influence cells and cause their death. Changes of GPXs, PRDXs and thioredoxin system gene transcripts by miRNA regulation leads to changes in cellular ROS and can affect cell survival (Table 3). In some cells such as NSCLC, breast cancer, or leukemia cells a decrease of antioxidant enzymes does not lead to apoptosis but has rather a pro-survival effect, perhaps reflecting differences in the optimal level of ROS required for specific cellular processes in different cell types.

## 6. Mutual Regulation of Elements of the Redox System

### 6.1. Changes of Cellular H_2_O_2_ Levels Are Accompanied by Changes in Levels of miRNAs and Their Targets

Exposure of cells to different oxidative stressors such as ionizing radiation, H_2_O_2_, or etoposide induces different cellular responses [152] and in many studies H_2_O_2,_ a relatively stable oxidant, has been used to study the different effects of increased ROS levels. In cells exposed to H_2_O_2_, the levels of multiple miRNAs that target transcripts of enzymes responsible for ROS/RNS production and neutralization were altered; however, the response to H_2_O_2_ could be different in different types of cells and miRNAs could be either down- or up-regulated after H_2_O_2_ treatment depending on H_2_O_2_ dose. For example, in ARPE-19 cells miR-23a which targets SOD2 and TXNRD1 is upregulated by H_2_O_2_ at concentrations up to 200 µM, but downregulated at higher concentrations [153] whereas in rat cardiomyocytes miR-181a which targets GPX1 is downregulated below 100µM H_2_O_2_ and upregulated above this concentration [116]. Similar dose-dependent effects were also observed for miRNAs targeting transcripts of other redox regulating enzymes including NOX, NOS, CAT, GPX, GSR, PRDX, TXN and TXNRD [83,116,154,155] as summarized in Table 4.

The increased levels of miRNAs which down-regulate the expression of antioxidant enzymes, observed after exposure to an oxidant, are rather counterintuitive. However, H_2_O_2_ plays a role of a signaling molecule and the observed changes may be elements of the establishment of H_2_O_2_ levels specific for the cell type and conditions. Inhibition of SOD would attenuate conversion of superoxide to H_2_O_2_, and in this way cells may prevent excessive H_2_O_2_ levels and enhance other, dependent on superoxide, pathways of the redox control system. Opposite to this inhibition of H_2_O_2_ neutralizers would enhance the H_2_O_2_ signaling.

**Figure 4:**
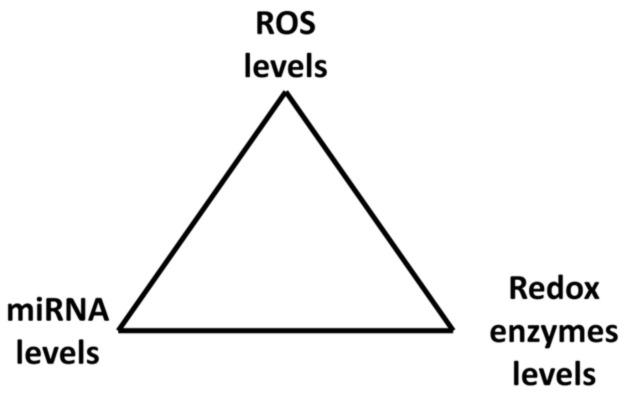
Mutual regulation of miRNAs and ROS through regulation of redox enzymes.

MiRNAs can be either down- or up-regulated after exposure to H_2_O_2_ depending on the H_2_O_2_ concentration. Therefore, miRNA levels depend on ROS concentrations in cells. ROS and miRNAs therefore create a kind of vicious circle or better a triangle where miRNAs affect ROS levels through ROS/RNS enzymes while ROS (H_2_O_2_) affect miRNAs levels; they are strictly connected and mutually influence each other. Together they create a system of feedback loops which may administrate cell responses to environmental conditions (Figure 4).

### 6.2. Unexpected Effects of Changes in Levels of ROS Producers and Neutralizers

One can find many examples where changes in the levels of ROS-producing enzymes, achieved by different methods in specific cell types, are accompanied by unexpected changes of the levels or activities of proteins participating in ROS neutralization. Table 4 shows examples of such non-intuitive responses of cells to an increase of ROS by increasing levels of miRNAs which target mRNAs for antioxidant enzymes.

In some cell types the levels of NOX are negatively correlated with the levels of ROS scavengers such as CAT, SOD, GPX and TXNL1 [59,62] and positively correlated with TXNIP levels (which increases ROS levels through inhibition of TXN) [67]. In experiments performed with mice spinal cord in which a decrease in NOX4 level (followed by a decrease of ROS) was induced, the increase in expression levels of the antioxidants GPX3 and TXNL1 was also observed [62]. Similarly, miR-320 overexpression and the subsequent NOX2 decrease was accompanied with increased CAT, SOD and GPX contents in ischemic mice cerebral neurons [59]. In human retinal micro-vascular endothelial cells the increase of NOX4 level obtained by a decrease of miR-590-3p level was accompanied by an increase of TXNIP which is an inhibitor of thioredoxins [67]. In the cells of rat brains, knockdown of NOX4 was accompanied by enhancement of GPX and SOD levels [163]. In airway smooth muscle cells, MnSOD expression was inhibited after increased expression of NOX4 [164]. In HUVECs loss of NOX4 reduced eNOS expression and NO production [97]. There are further examples of mutual NOX and NOS influences. In VSMCs NOX2 overexpression and increased ROS production led to a significant increase of NOS protein, and MnSOD protein level was also increased [165]. A contrary effect was seen in studies of Gregg et al. where CAT and SOD were downregulated after inhibition of NOX4 [166] and in work of Jeong et al. where SOD and GPX were decreased after NOX4 knockdown [167].

### 6.3. The Same miRNAs May Regulate the Expression of Both ROS-Producing and ROS-Neutralizing Enzymes

**Figure 5:**
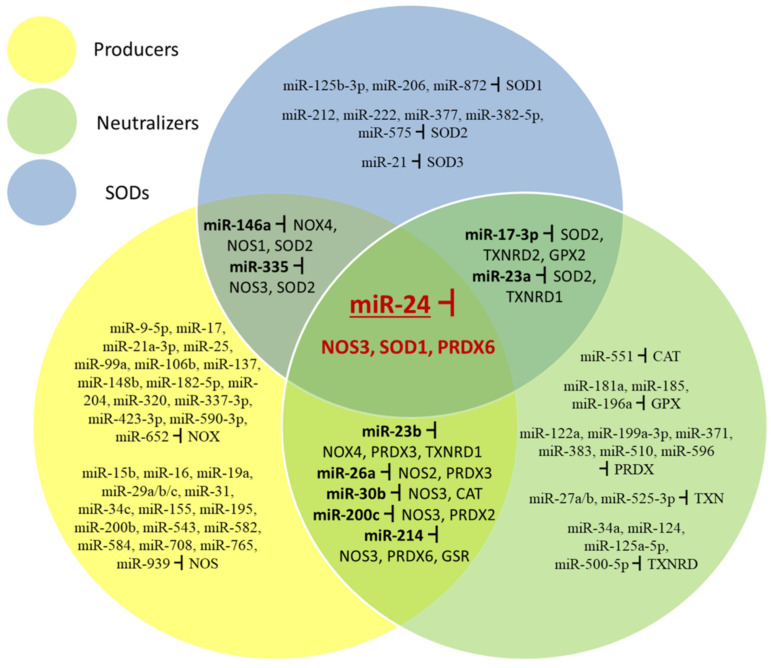
miRNAs which target different parts of the redox control system (ROS-producing, ROS-neutralizing, and SOD enzymes).

The data reviewed here show that responses of ROS/RNS systems in cells are at least in part regulated by miRNAs. Surprisingly, the same miRNA may target mRNAs for proteins which have opposite effects. Figure 5 summarizes miRNAs which target mRNAs coding for enzymes producing and neutralizing ROS. SOD enzymes are treated separately because they neutralize superoxide but at the same time produce H_2_O_2_ which is an oxidant. While most miRNAs target only ROS producers, ROS neutralizers, or SODs, some target both mRNAs for enzymes producing and neutralizing ROS, and one miRNA which targets all three types of redox enzyme.

MiR-17-3p can target SOD2, TXNRD2 and GPX2 and miR-23a SOD2 and TXNRD1 transcripts affecting elements of conversion and neutralization of H_2_O_2_. MiR-23b can target NOX4, TXNRD1 and PRDX3; miR-26a NOS2 and PRDX3; miR-30b NOS3 and CAT; miR-200c NOS3 and PRDX2; miR-214 NOS3, PRDX6 and GSR transcripts affecting producing and neutralizing steps of the redox system. MiR-146a targets NOX4, NOS1 and SOD2 and miR-335 targets NOS3 and SOD2 transcripts which affect superoxide production and conversion. One miRNA (miR-24) affects all three elements of the ROS/RNS system, NOS3, SOD1, and PRDX6 transcripts.

Changes of cellular concentrations of miRNAs which may simultaneously downregulate expression of ROS producers and neutralizers must be connected to circuits which specifically regulate death and survival in particular conditions. Depending on the ROS level, regulation may be organized in positive or negative feedback loops and thus cells adjust their redox environment to achieve optimal levels for specific cellular processes.

## 7. Role of miRNAs as Regulators of Redox Balance in Cancer Development

It is well known that redox balance is impaired and that ROS levels are persistently high in cancer cells. These increased levels may result in activation of oncogenes and oncogenic signals [168], and some cancer cell lines can produce constitutively high levels of ROS which result in their increased proliferation [43]. To cope with excess levels of ROS, cancer cells have developed systems by which they adapt through activation of antioxidant pathways and development of efficient mechanisms for neutralization of ROS An increased level of antioxidants in cells may promote tumor growth and metastasis; a small increase enhances metastasis of melanoma or progression of lung cancer in mice [169,170]. Each cell type has its own specific optimal levels of ROS which allow to regulate proliferation and other processes. This can be obtained through adjustment of the levels of enzymes responsible for production or neutralization of ROS and may be connected to miRNA levels; for example, miR-99a and miR-137 which target ROS producers such as NOX are downregulated in adenocarcinoma [55] and prostate cancer [65], respectively and miR-212 which targets SOD2 is decreased in colorectal cancer, causing elevation of SOD2 levels [103]. Enzymes which are responsible for H_2_O_2_ neutralization can be either down- or upregulated by miRNAs. MiR-510 and miR-596 target PRDX1, and miR-510 is elevated in breast cancer [120] whereas miR-596 is downregulated in gastric cancers [121]. PRDX3 levels are elevated in prostate cancer and reduced by miR-23b [126]. Changes of PRDX3 are also observed in medulloblastoma where miR-383 is underexpressed [127]. These varying effects show that miRNAs may be responsible for pathological states in cells through influencing redox systems and that adjustment of ROS levels is achieved in different ways in different cell types.

The multiple examples of miRNAs which can directly target genes connected to redox equilibrium indicate that miRNAs are important modulators of redox balance. Apparently paradoxical phenomena sometimes occur and remain to be understood, such as when oxidative stress induces an increase in the levels of miRNAs which target and suppress expression of genes coding for enzymes which neutralize ROS, or when the same miRNA targets genes coding for enzymes with opposing functions in redox regulation. Taken together, the studies reviewed here indicate the existence of very sensitive, strictly regulated, and conserved regulation mechanisms which allow cells to survive when exposed to chronic oxidative stress.

## Figures and Tables

**Table 1:** Cellular effects of an increased level of miRNAs which target transcripts for enzymes producing ROS and RNS. MiRNAs in grey rows show an opposite effect on cell survival to those in white rows.

Targeted Transcript and miRNAs	Cellular Effect	Cell Types	References
**NOX2** ┣ miR-320, miR-652	Pro-survival	Cerebral neurons, brain tissues of rats	[59,60]
**NOX4** ┣ mir-23b, miR-182-5p, miR-423-5p, miR-590-3p	Pro-survival	Mouse spinal cord, human lens epithelial cells, mouse podocytes, human retinal microvascular endothelial cells	[62,63,66,67]
**NOX4** ┣ miR-99a, miR-137	Pro-apoptotic	Lung adenocarcinoma, prostate cancer	[64,65]
**NOS3** ┣ miR-24, miR-155	Pro-survival	VSMCs, cardiomyocytes, cardiac fibroblasts	[76,82,84]
**NOS3** ┣ miR-24, miR-31-5p, miR-155, miR-200c, miR-335, miR-543, miR-584	Pro-apoptotic	HUVECs, prostate cancer, trophoblasts, human microvascular endothelial cells	[77,78,80,81,82,83,84]
**NOSIP** ┣ miR-372	Pro-apoptotic	Human neural stem cells	[96]

**Table 2:** Effects of increased levels of miRNAs which target transcripts of SOD enzymes. MiRNAs in grey rows have an opposite effect on cell survival to those in white rows.

Targeted Transcript and miRNAs	Cellular Effect	Cell Types	References
**SOD2** ┣ miR-17-3p, miR-23a, mir-146a, miR-212, miR-575	Pro-apoptotic	Rat PC12 adrenal gland cells, villi cells, prostate cancer, ARPE-19 cells, cardiomyocytes, colorectal cancer	[98,99,100,101,102,103,112]
**SOD2** ┣ miR-222, miR-382-5p	Pro-survival	Oral tongue squamous cell carcinoma, primary myelofibrosis CD34+ cells	[104,106]
**SOD3**┣ miR-21	Pro-survival	Human bronchial epithelial cells	[109]

**Table 3:** Effects of increased levels of miRNAs which target transcripts of H_2_O_2_-regulating enzymes. miRNAs in grey rows show opposite effects on cell survival to those in white rows.

Targeted Transcript and Increased miRNAs	Cellular Effect	Cell Types	Reference
**GPX1** ┣ miR-181a	Pro-apoptotic	Cardiomiocytes	[116]
**GPX2** ┣ miR-17-3p	Pro-apoptotic	Prostate cancer	[98,99]
**GPX3** ┣ miR-196a	Pro-survival	NSCLC	[119]
**PRDX1** ┣ miR-510	Pro-survival	Breast cancer	[120]
**PRDX1** ┣ miR-596	Pro-apoptotic	Gastric cancer	[121]
**PRDX2** ┣ miR-122a, miR-200b, miR-200c	Pro-apoptotic	Hepatocellular carcinoma, colorectal cancer, lung cancer	[122,123,124]
**PRDX3** ┣ miR-383	Pro-apoptotic	Medulloblastoma	[127]
**PRDX6** ┣ miR-199a-3p	Pro-survival	Leukemia	[129]
**PRDX6** ┣ miR-24-3p, miR-371	Pro-apoptotic	Gastric cancer PC9 xenograft mouse model	[128,131]
**TXN1** ┣ miR-525-3p	Pro-survival	EA, HeLa, RPE, U2-OS	[132]
**TXNRD1** ┣ miR-23a/b, miR124, miR-125a/b-5p	Pro-apoptotic	Skeletal muscle, lung cancer, head and neck cancer, hepatocellular carcinoma, human pigment epithelial cells, prostate cancer	[98,99,100,134,135,137,138]
**TXNRD2**┣ miR-17-3p	Pro-apoptotic	Mesangial cells	[107]
**TXNIP** ┣ miR-20b, miR-135a, miR-152, miR-224, miR-224/452, miR-373, miR-411-5p	Pro-survival	HUVECs, microglial BV2 cells, pancreatic cancer, melanoma, breast cancer, NSCLC	[143,144,146,148,149,150,151]
** **TXNIP** ┣ miR-17 miR-148a	Pro-apoptotic	Myocardial cells of diabetic mice, hepatocytes	[141,147]
** miR-17 and miR-148a levels were decreased, and thus a pro-apoptotic effect is expected.			

**Table 4:** H_2_O_2_-induced changes of levels of miRNAs which regulate enzymes of redox systems.

miRNA and Targeted Transcripts	H_2_O_2_ Effect on miRNA Level	H_2_O_2_ Dose	Cell Type	Ref.
**miR-17-3p** ┫ SOD2, GPX2, TXNRD2	Up Down	0–100 µM 200 µM	ARPE-19 cells	[100]
**miR-21** ┫ SOD3	Up	0–200 µM	VSMCs	[156]
**miR-23a** ┫ SOD2, TXNRD1	Up Down	100–200 µM 300–500 µM	ARPE-19 cells	[153]
**miR-24** ┫ NOS3, SOD1, PRDX6	Up	400 µM	Human lens epithelial cells	[157]
**miR-30b** ┫ NOS3, CAT	Up	200 µM	ARPE-19 cells	[113]
**miR-122** ┫ PRDX2	Down	600 µM	ARPE-19 cells	[158]
**miR-135a** ┫ TXNIP	Up	0–1 mM	Rat cardiomyoblasts	[159]
**miR-146a** ┫ NOX4, NOS1, SOD2	Up	0–200 µM	Rat PC12 cell from adrenal gland	[112,160]
**miR-155** ┫ NOS3	Up	0–500 µM	VSMCs	[161]
**miR-181a** ┫ GPX1	Down Up	0–100 µM 200–600 µM	Rat cardiomyocytes H9c2	[116]
**miR-200c** ┫ NOS3, PRDX2	Up	400 µM	Normal human liver LO2 cells	[162]
	Up	200 µM	HUVECs	[83]
**miR-214** ┫ NOS3, PRDX6, GSR	Up	0–100 µM/L	Cardiomyocytes	[154]
	Up	0–600 µM/L	Skeletal myoblasts	[155]
**miR-500a** ┫ TXNRD1	Up	0–10 µM	Breast cancer MCF-7 cells	[139]
